# “*Poverty is a social issue*,* not a mathematical problem*”: examining the lessons for beneficiary identification from implementation of the UHC indigent program in Kenya

**DOI:** 10.1186/s12939-026-02767-5

**Published:** 2026-01-29

**Authors:** Beryl Maritim, Rahab Mbau, Anita Musiega, Anne Musuva, Beatrice Amboko, Benjamin Tsofa, Caitlin Mazzilli, Ileana Vilcu, Ethan Wong, Felix Murira, Jacinta Nzinga, Matt Boxshall, Peter Mugo, Rose Nabi Deborah Karimi Muthuri, Wangari Ng’ang’a, Nirmala Ravishankar, Edwine Barasa

**Affiliations:** 1 Health Economics Research Unit, KEMRI-Wellcome Trust Research Programme, Nairobi, Kenya; 2 Gates Foundation, Seattle, WA USA; 3 Health Systems insight, Washington, DC USA; 4 Health Systems Insight, Nairobi, Kenya; 5 Center for Tropical Medicine and Global Health, Nuffield Department of Medicine, University of Oxford, Oxford, UK

## Abstract

**Background:**

Kenya rolled out a UHC indigent program aimed to expand financial protection and health service access for poor households through subsidized health insurance under the national insurer, National Health Insurance Fund (NHIF). As Kenya transitions to a new social health insurance framework under the Social Health Authority (SHA), understanding the implementation experience of the UHC indigent program is critical for informing the roll out of SHA’s indigent program.

**Methods:**

We conducted a qualitative process evaluation of the UHC indigent program using document reviews, semi-structured interviews with 23 key informants from national and county health authorities, development partners, and implementing actors, complemented by a validation workshop with 57 stakeholders. Our analysis was guided by Moore et al.‘s process evaluation framework and Wu et al.‘s policy capacity lens, examining implementation fidelity and capacities at multiple levels.

**Results:**

The program’s implementation deviated from its original centralized design, with counties exerting control over beneficiary identification due to national data gaps, incomplete rollout of the Harmonized Testing Tool, and political and operational constraints. Variations in targeting methods, reliance on under-resourced community health actors, and delays in biometric registration contributed to partial enrolment, limited access, exclusion errors, and mistrust. Although some counties reported increased service utilization, this was limited by unregistered dependents and lack of beneficiary awareness. Stakeholders expressed concern over SHA’s use of proxy means testing for identifying the poor, citing risks of exclusion, manipulation, and failure to capture locally constructed definitions of poverty.

**Conclusion:**

Kenya’s experience demostrates the need to align national targeting frameworks with local realities, invest in policy capacity across stakeholders, and prioritize community validation and communication in subsidy programs. As SHA rolls out a new indigent program, these lessons offer critical guidance for enhancing fidelity, equity, and accountability.

## Introduction

Many low- and middle-income countries (LMICs) have embraced Universal Health Coverage (UHC), implementing various health insurance models, including social health insurance (SHI), to enhance financial access to healthcare for their populations [15, 43]. In alignment with the Sustainable Development Goals, UHC is rooted in the need to shield individuals from the financial hardships associated with out-of-pocket (OOP) health expenditures, mitigating the risk of catastrophic and impoverishing healthcare payments [27]. However, there is growing concern that SHI models, particularly in Africa, often leave out the poorest and most vulnerable populations due to contributory requirements, weak identification systems, and limited fiscal space to subsidize premiums [8, 11, 14]. These exclusions threaten to widen inequities in access and undermine the core goals of UHC. In response to equity concerns, many Health insurance programs are accompanied by efforts to provide coverage to poor and vulnerable households through government sponsored health insurance Health Insurance subsidies.

Subsidies can take various forms, including premium subsidies that lower the cost of insurance coverage, direct subsidies to healthcare providers to offset the cost of services for targeted groups, and supply-side subsidies aimed at improving healthcare infrastructure and service delivery in underserved areas [1]. HISPs are financial intervention designed to reduce barriers to healthcare access, particularly for populations unable to afford the full cost of insurance premiums or healthcare services [31, 45]. Subsidies are particularly relevant for countries with a large informal sector-often accounting for over 70% of the workforce-where traditional employment-based health insurance models are less viable [29].

Research in countries such as Ghana, India, and Indonesia have found that premium subsidies can boost enrolment in health insurance programs, particularly among low-income and informal sector populations who would otherwise be unable to afford coverage [13, 23, 30, 35]. By reducing the financial barriers to obtaining health insurance, subsidies can increase access to healthcare and improve financial risk protection for vulnerable groups [23].

Evidence from health insurance subsidy suggests that the way these programs are designed can have a significant impact on enrolment rates and utilization patterns. This includes targeting mechanisms to identify and enrol eligible beneficiaries, the level of the subsidy offered, the methods for delivering subsidies (e.g., direct premium subsidies, vouchers), the benefit entitlements, and efforts to promote awareness and understanding of the program [23]. Designing and implementing effective HISPs in LMICs presents several challenges that require careful consideration of factors such as equity, stakeholder engagement, and sustainability to ensure the long-term success and impact of these programs [6, 13]. Kenya’s pursuit of UHC was, until recently, anchored in the National Hospital Insurance Fund (NHIF), which served as the country’s national social health insurance (SHI) scheme from its establishment in 1966 until its replacement in 2023. Over the years, NHIF expanded its mandate and benefit package but continued to face challenges of limited coverage among informal sector workers, weak mechanisms for subsidizing indigent households, and inefficiencies in purchasing health services [19]. In response, the government enacted a new set of reforms through the Social Health Insurance Act (SHIA) 2023, which seeks to transform Kenya’s social health insurance system [44]. The Act establishes the Social Health Authority (SHA) as the successor to NHIF and introduces three funds-the Primary Healthcare Fund, the Emergency, Chronic and Critical Illness Fund, and the Social Health Insurance Fund (SHIF)-to enhance risk pooling, strengthen strategic purchasing, and expand coverage for poor and vulnerable populations through tax-financed subsidies. The SHI reforms are also situated within Kenya’s broader national development blueprints-building on Kenya Vision 2030 , the Big Four Agenda, and the current government’s Bottom-Up Economic Transformation Agenda (BETA*)* -which collectively position UHC as a key pillar of socio-economic transformation and inclusive growth ([16, 17]; Parliamentary Service Commission, 2023).

**Fig. 1:**
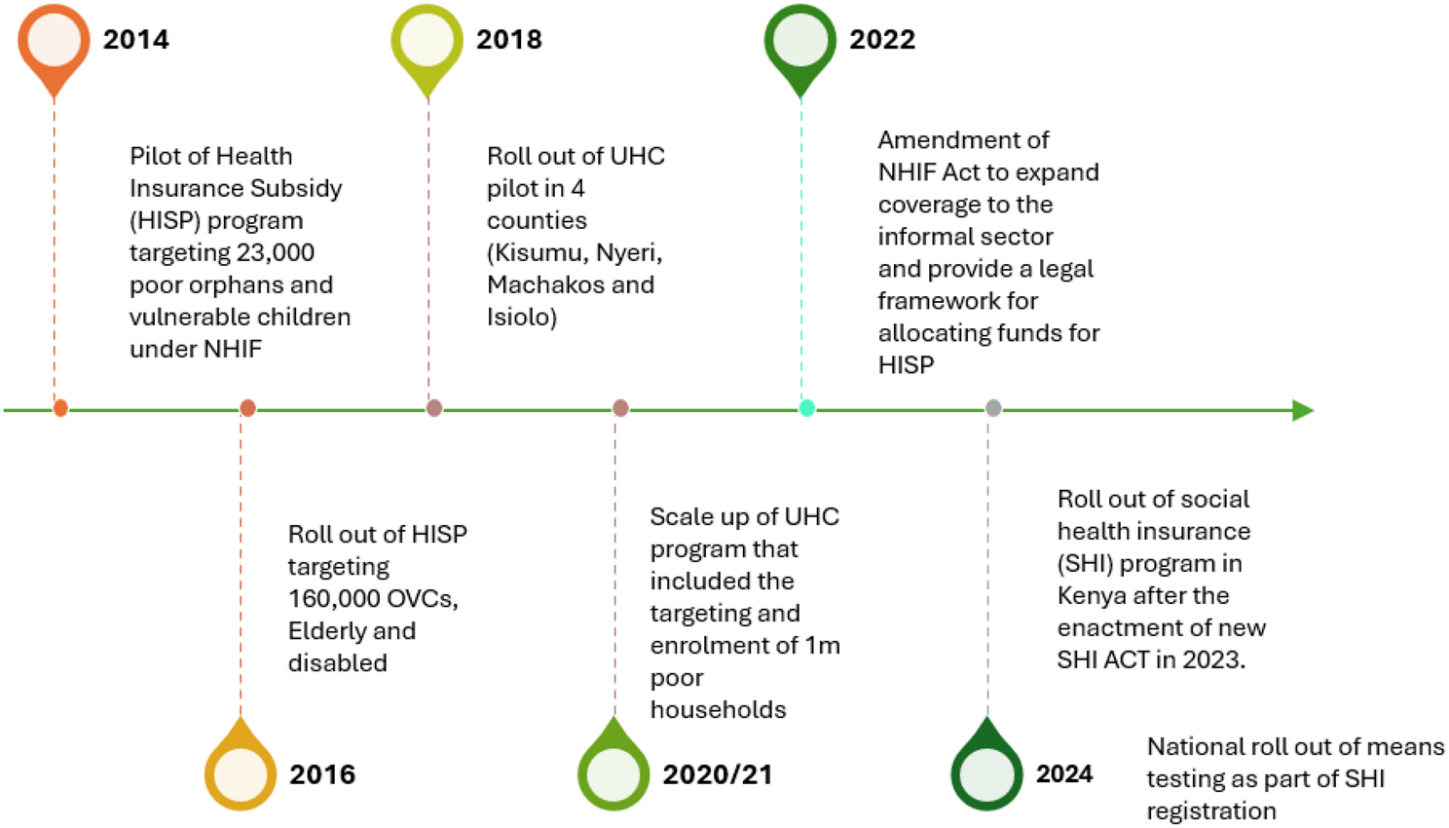
Timeline of health insurance subsidy program evolution in Kenya

Kenya’s current indigent targeting efforts under the SHA build on a decade of earlier initiatives to expand health insurance coverage among poor households. The first, the Health Insurance Subsidy Program (HISP), was launched in 2014 and scaled up in 2016 to cover approximately 160,000 Orphans and Vulnerable Children (OVC), people living with severe disabilities, the elderly (over 65 years) and poor households [7, 21]. Beneficiaries of the program were identified by the ministry of labour and social protection (MOLSP). However, evaluation findings revealed significant targeting errors, with about 65% of beneficiaries drawn from the wealthiest quintiles, limiting the program’s equity impact [7]. Building on lessons from HISP and the 2018 UHC pilot program implemented in four counties (Kisumu, Nyeri, Isiolo, and Machakos), the government rolled out the UHC indigent program in 2020/21 (Fig. 1).

This paper presents the findings of a process evaluation of the design and implementation of Kenya’s UHC indigent scale-up program (2020/21–2022). We examine the implementation process, exploring how the program was designed, coordinated, and executed, the capacities of key stakeholders, and the contextual and political factors influencing fidelity to the intended design. The findings are particularly timely as Kenya transitions from the NHIF to the new SHA. The lessons drawn from the UHC indigent program-its design strengths, coordination challenges, and implementation bottlenecks-offer critical evidence to inform the rollout of the new indigent identification and subsidy arrangements under the SHA framework.

**Fig. 2:**
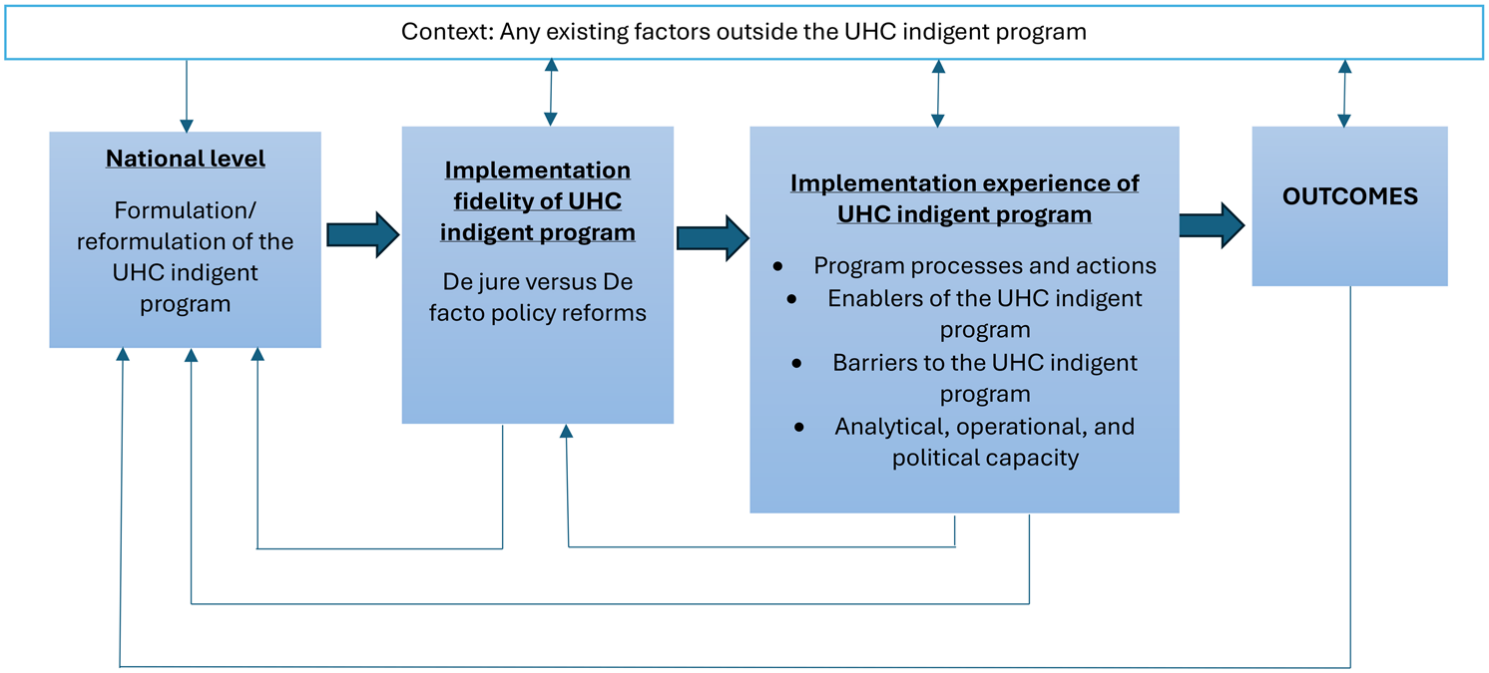
Study conceptual framework (Authors, 2025)

## Methodology

### Conceptual framework

To examine the implementation experience of the UHC indigent program, we conducted a process evaluation. This evaluation aimed to draw lessons that could inform the roll out of national subsidy program for the poor under SHA. Our process evaluation framework (Figure 1) was adapted from [36]). We first explored the emergence of the UHC indigent program. We then described and examined the design, activities, and processes, as well as the fidelity of the implementation. We also explored the experiences of relevant stakeholders, including national and county-level policymakers and implementers. To provide a comprehensive analysis, we integrated Moore et al.‘s framework with concepts of policy capacity, which refers to the skills and competencies needed to carry out a policy function [46]. Drawing on Wu et al., we assessed three dimensions of policy capacities that influenced policy implementation: analytical policy capacities, which are the skills and competencies required to develop technically sound strategies to support the fulfilment of policy reform goals; operational policy capacities, which are the skills and competencies required to align resources with the goals of the policy to enable implementation; and political policy capacities, which are the skills and competencies required to identify, mobilize, and strengthen political support for policy actions. These policy capacities were assessed across the individual, organizational, and system levels of the policy environment. By examining the UHC pilot implementation experience through this lens, this process evaluation helped to identify areas for improvement in future UHC programs.

### Study context

Kenya’s health system operates within a devolved governance structure, established under the 2010 Constitution, which transferred responsibility for health service delivery to 47 county governments, while the national government retained roles in policy formulation, regulation, and national referral services [34]. The system is mixed, comprising public, private-for-profit, and faith-based (mission) facilities that collectively deliver health services nationwide. As of 2023, Kenya had approximately 14,403 health facilities, with 46.0% public, 46.9% private-for-profit, and 7.1% faith-based facilities [22]. Of these, only 3,858 facilities were contracted by the NHIF-58.5% public, 33.5% private, and 8.0% faith-based-to provide services under its benefit packages, including the Supa Cover scheme.

### Study design and participants

We conducted a qualitative study using document reviews, semi-structured interviews and a national stakeholder workshop to explore the development and implementation of UHC indigent program in Kenya. A total of 23 participants were interviewed, drawn from various health system stakeholders, including national-level bureaucrats, development partners, implementing organizations, civil society organizations, academia, and representatives from the NHIF and Kenya Healthcare Federation (KHF). Implementing partners refer to non-governmental or non-profit organizations funded by development partners or global health programs-such as PEPFAR, USAID, or the Global Fund-to provide technical and operational support to government agencies in implementing specific program activities.

We also interviewed county health officials, community health liaisons as well as community health volunteers (now known as promoters) to understand the implementation experience of the program at the county level in two purposively selected counties-Kisumu and Kiambu. Kiambu is in central Kenya and is mostly peri-urban population while Kisumu is in the Western part of Kenya with a mix of rural and urban population. Table 1 summarizes the county profiles against national estimates.

**Table 1:** Study counties profiles

	Kisumu	Kiambu	National
Population 2019 census (n)	1,155,574	2,417,735	47,564,296
Poverty rates (%)	36.3	20.5	38.6
Overall Health insurance coverage (%)	18	39.1	26.3
Average household size (n)	3.8	3.0	3.9
Criteria considered in selection	One of the four UHC pilot counties	Relatively higher socio-economic status and peri-urban County	

Source: [24-26]

Snowball sampling was used alongside purposive sampling to identify additional participants with relevant knowledge of the UHC indigent program. Initial key informants were purposively selected from national and county governments, NHIF, and development partners. During interviews, respondents referred the research team to other individuals with first-hand involvement in program design and implementation, enabling inclusion of actors who might not have been initially identified through formal lists (Table 2). Snowball sampling was then used to identify additional participants through referrals from initial interviewees.

**Table 2:** Summary of study participants

Participant category	Female	Male	Number
National policy makers- Ministry of Health (MOH) bureaucrats	1	3	4
County stakeholders (2 purposively selected counties)	4	3	7
Development partners	0	3	3
NHIF/SHA	1	2	3
Implementing partners	1	3	4
Civil Society Organization (CSO)	0	1	1
Academia	0	1	1
Total	7	16	23

We collected data using a semi-structured topic guide developed from the components of the study’s conceptual framework (Fig. 2). The guide was designed to ensure consistent coverage of relevant themes while allowing flexibility to probe emerging issues during interviews. Questions explored the rationale and motivations for establishing the indigent program (“Why was the UHC indigent program developed, and what problems was it intended to address?”), the processes and capacities involved in its implementation (“Which actors were involved in identifying and registering indigent households, and how adequate were their resources and capacities?”), and perceptions of its performance (“How satisfied were stakeholders with the procedures used to identify beneficiaries and the services received under the program?”). Probes were used throughout to elicit detailed accounts of decision-making, coordination mechanisms, contextual influences, and lessons learned. All interviews were conducted by BM and RM in the offices or workplaces of the participants, providing a setting that supported open discussion. Each interview lasted approximately 45 min to 1 h. These were audio-recorded with participant consent and transcribed verbatim for analysis.

### Document review

To complement and triangulate the interview data, a targeted review of relevant policy, legislative, and program documents was undertaken. The aim was to contextualize participants’ perspectives, verify the design and implementation details of the UHC indigent program, and situate the findings within Kenya’s evolving UHC and social protection landscape. Table 3 presents the key documents reviewed.

**Table 3:** Documents included in review

Category	Document Title	Year	Source	Purpose of Review
A. Statutes and Regulations	Social Health Insurance Act and Regulations	2023–2024	Government of Kenya	Defined the legal and institutional framework for the Social Health Authority and indigent program
	NHIF Act and NHIF Amendment Act	1998, 2002	Government of Kenya	Provided the legislative foundation for health insurance and the transition to SHA
	Kenya Social Protection Policy	2023	Ministry of Labour and Social Protection	Outlined national principles and mechanisms for social protection targeting the poor
B. National Reports and Strategies	Vision 2030	2008	Government of Kenya	Set out the national development agenda, including health sector and UHC goals
	Kenya Kwanza Manifesto	2022	Government of Kenya	Presented political commitments to universal health coverage and social welfare
	The Big Four Agenda – Frequently Asked Questions on the Universal Health Care Program	2018	Government of Kenya	Provided background on UHC priorities under the Big Four Agenda
	10th Annual President’s Report on National Values and Principles of Governance	2023	Government of Kenya	Reported national UHC efforts including the numbers of indigent households identified, implementation constraints and recommendations for improvements.
C. Program and Policy Documents	Health Financing Expert Review Panel (HEFREP) Report – *The NHIF We Want*	2023	Ministry of Health / HEFREP	Provided analysis and recommendations on transforming NHIF into a strategic purchaser
	Health Sector Report, Medium-Term Expenditure Framework (MTEF) 2024/25–2026/27	2023	Ministry of Health	Offered information on health budget allocations and sector priorities
	Sector Working Group Report, MTEF 2023/24–2025/26	2023	Ministry of Health	Provided insight into health sector resource planning and fiscal projections
	Harmonized Testing Tool	2023	Ministry of Labour and Social Protection	Outlined the operational procedures for identifying indigent households
	Evidence Brief: *Lessons from the Kenya National Social Health Insurance Reforms and Implementation*	2022	AFIDEP	Summarized evidence on social health insurance reform implementation and lessons learned
	*A Harmonised Proxy Means Test for Kenya’s National Safety Net Programme*	2021	University of Manchester (Juan M. Villa)	Provided methodological background on means testing and targeting systems
D. Other Reference Materials	*NHIF and Financing of UHC*	2021	Kenya Yearbook Editorial Board	Summarized NHIF’s role in Kenya’s health financing system and UHC efforts
	Media reports on the UHC indigent program	2023–2024	Various national media	Provided additional information on rollout progress, and implementation challenges

### Data analysis

Data was analyzed thematically using the six-step approach proposed by Braun and Clarke (2006). First, the research team immersed themselves in the data by reading and re-reading the transcripts (Step 1). Second, a list of deductive codes was developed based on the concepts from the study’s conceptual framework (Step 2). The initial list of deductive codes was generated from the main domains of the study’s conceptual framework, which combined Moore et al.’s [36] Process Evaluation Framework and Wu et al.’s [46] Policy Capacity Framework. These frameworks informed the coding structure by identifying key dimensions related to context, implementation processes, mechanisms, and outcomes [36], and the types of capacities-analytical, operational, and political-across individual, organizational, and system levels [46].The codebook was refined iteratively as transcripts were reviewed, with the two coders comparing interpretations and resolving discrepancies through discussion to ensure consistency and rigor.

In the third step, similar codes were grouped into themes by identifying patterns across the coded data (Step 3). Fourth, themes were reviewed for internal consistency and coherence with the coded extracts to ensure they accurately reflected the data (Step 4). In the fifth step, the finalized themes were systematically applied to the entire dataset, with supporting quotes and excerpts identified for each theme (Step 5). In the final step, the findings were synthesized and interpreted in relation to both empirical and theoretical literature. The interpretation was guided by the two study frameworks: Moore et al.’s [36] Process Evaluation Framework and Wu et al.’s [46] Policy Capacity Framework. The Moore framework helped organize findings around the contextual factors, implementation processes, mechanisms of change, and emerging outcomes of the indigent program. The Policy Capacity framework provided an analytical lens to assess the analytical, operational, and political capacities that enabled or constrained implementation across individual, organizational, and system levels. Findings were then compared with empirical literature on health financing and indigent targeting reforms in sub-Saharan Africa to situate Kenya’s experience within a broader body of evidence and to explain how contextual, institutional, and actor dynamics shaped implementation outcomes (Step 6). Coding was conducted by two members of the research team (BM and RM), who independently reviewed and compared the coded transcripts, discussing and resolving discrepancies through consensus to ensure analytical rigor.

## Results

The results are organized around five thematic areas that align with the study’s analytical framework reflecting the evolution of the UHC indigent program from design to implementation. The first section outlines the policy rationale and contextual factors underpinning the program’s development. The second examines the intended design and implementation fidelity, contrasting the program’s formal (de jure) design with its actual (de facto) implementation. The third section assesses stakeholder roles and capacities using the analytical, operational, and political dimensions of the Policy Capacity Framework. The fourth presents program outcomes, including enrolment progress and biometric registration performance. The final section highlights implementation challenges, unintended consequences, and lessons learned to inform the ongoing rollout of the SHA indigent program. A summary of the key findings across these thematic areas is provided in Table 4 below.

**Table 4:** Thematic areas and key findings

Thematic area	Key findings
Programme development rationale	The programme was informed by national commitments to financial risk protection, with a policy shift from input-based financing to insurance-based subsidies to improve sustainability, efficiency, and accountability.
Programme design and intended implementation	The UHC indigent programme was designed as a nationally targeted insurance scheme covering one million households (approximately 4.4 million individuals), implemented through NHIF and relying on MOLSP tools (ESR and HTT) for standardised identification.
De facto implementation (fidelity)	Implementation diverged from design due to decentralisation of beneficiary identification, data-sharing constraints, and county discretion, resulting in heterogeneous targeting approaches and variation across counties.
Stakeholder roles and capacity	MOH provided strategic oversight with limited enforcement capacity; NHIF managed enrolment and benefits administration; MOLSP had strong analytical capacity but limited operational engagement; counties led identification with uneven capacity; CHVs and local actors played central roles but lacked adequate tools and support.
Operational challenges	Fragmented data systems, delayed fund disbursements from treasury to NHIF, constrained CHV logistics, and political pressures undermined implementation fidelity. Counties with greater facility-level financial autonomy experienced fewer disruptions.
Programme outcomes	A total of 882,291 households (88% of target) were enrolled, but only 382,000 (48%) completed biometric registration; some counties filled as little as 50% of allocated quotas. Increased utilisation was reported where biometric registration was higher.
Unintended consequences	Inclusion and exclusion errors, overlapping subsidy programmes, and political influence weakened equity and eroded trust in the programme.
Lessons for SHA rollout	Stakeholders supported more standardised targeting approaches, including PMT, but raised concerns about feasibility, gaming risks, and exclusion of the poorest, underscoring the need for transparency and community validation.

### Program development rationale

Policy makers reported that the UHC indigent program was conceived as part of Kenya’s broader national commitment to expand financial risk protection (FRP) under Vision 2030 and the Big Four Agenda.

*The biggest issue was that poor households were being pushed further into poverty by healthcare costs. The program was meant to stop this cycle* (KII_C1_05).

Experience from earlier UHC efforts further shaped the design and implementation of the program. In particular, the 2018 UHC pilot relied on an input-based financing model, where government supplied commodities and operational inputs directly to facilities. Although this approach facilitated rapid service expansion during the pilot, policymakers assessed it as fiscally difficult to sustain. These concerns reinforced the need for a more scalable financing mechanism, prompting a shift toward an insurance-based approach.

*The UHC pilot showed us that input-based financing wasn’t working as intended. We needed a model that could scale*,* and that’s where the shift to insurance came in* (KII_NHIF_02).

In addition, the UHC indigent programme was informed by Kenya’s existing experience with subsidised health insurance for vulnerable groups. At the time of rollout, approximately 374,500 individuals were already receiving fully subsidised NHIF coverage through Inua Jamii–linked Health Insurance Subsidy Programmes targeting older persons, orphans and vulnerable children, and persons with severe disabilities. Respondents indicated that the existence of these programmes, together with the administrative systems used to manage them, shaped expectations that indigent coverage could be expanded nationally by building on established social protection infrastructure.

Legislative reforms were also initiated to support this trajectory. The NHIF Amendment Act (2022) redefined NHIF’s mandate and formalised the use of public funds to subsidize premiums for indigent households, providing the legal basis for enrolling poor households into Supa Cover with full government financing.

### Key actors and their roles in design and implementation

Several stakeholders were involved in the design and implementation of the UHC indigent program including: the MOH, MOLSP, NHIF, Council of Governors (COG), County Governments, Civil Society Organizations, Village elders and Community Health Volunteers (CHVs). The MOH was charged with the formulation and coordination of the UHC policies, overseeing the overall implementation of the program. Its role included ensuring that indigent households were identified and enrolled in the program, while also coordinating with stakeholders at both national and county levels.

*The Ministry of Health had to ensure that all stakeholders*,* including county governments and development partners*,* were involved in the planning and implementation process* (KII_MOH_03).

MOLSP is the national institution responsible for social protection policy, within which social health protection is defined as a core pillar. Through its Department of Social Protection, MOLSP manages national social protection programmes and maintains the Enhanced Single Registry (ESR), a socioeconomic database of vulnerable households. The ESR includes beneficiaries of earlier NHIF-managed HISPs - those supported under Inua Jamii, which targets older persons, orphans and vulnerable children, and persons with severe disabilities.

NHIF was responsible for managing the insurance coverage for the indigent households identified under the program. NHIF’s role was to verify household data provided by County governments and ensure that beneficiaries were enrolled in the system. NHIF had successfully ran a pilot and scale up of the HISP and was expected to utilize the same processes working with the MOLSP to scale up the national UHC indigent program. NHIF’s operational role was therefore essential in managing the household registration and claims process.

*NHIF played a key role in ensuring that once households were identified*,* they were enrolled in the insurance program and could access services without delay* (KII_NHIF_02).

Development partners, such as the World Health Organization (WHO), the World Bank, UNICEF, and Clinton Health Access Initiative (CHAI), provided technical and financial support throughout the program’s formulation and implementation. These partners helped realign existing projects to support UHC, offering both financial resources and expertise.

*Development partners helped us a lot*,* especially in providing the technical support needed to develop policies and realign their projects to support UHC.”(KII_MOH_03).*

The COG - the coordinating body representing Kenya’s 47 county governments- played a key role in linking national and county stakeholders, convening consultations, and facilitating the rollout of the program in the counties.

Lastly, County Governments were tasked with identifying and registering indigent households. National operations and tools were to be cascaded to the counties through the COG for a streamlined implementation of the program. In addition, County governments managed various complementary social protection programs that included local HISPs. CSOs were engaged mainly in advocacy, community sensitization, and accountability efforts. At the community level, Village elders and CHVs played a central role in identifying indigent households and relaying the lists if identified beneficiaries to the county governments.

### Program design and implementation fidelity

#### Intended program design- dejure design

The UHC indigent programme was conceived as a multi-stakeholder initiative involving national and county institutions. Its design envisaged the use of standardised national tools to support county-level identification of indigent households, drawing on processes previously applied under health insurance subsidy programmes for older persons, orphans, and persons with disabilities. Central to this approach was the Harmonized Targeting Tool (HTT), which applies proxy means testing and was intended to be complemented by community-based verification involving local administrative structures.

*The Department of Social Protection had the mandate to identify indigents using their data*,* and this was supposed to be standardized across counties”(KII_Do_02).*

Beneficiary identification was to be anchored in the Enhanced Single Registry (ESR), with verified household data integrated into NHIF enrolment systems to support consistent targeting across counties

*The single registry was for purposes of having a database whereby if there’s any program that is targeting vulnerable persons*,* you would easily find a database of vulnerable persons that that is already accessible and*,* it would be easy to start off your programs because you’ve identified the vulnerable persons. (KII_IP_05)*

*There was a discussion of how households should be identified by expanding the use of the existing tools that have been used in the HISP*,* but because the identification was done by the Ministry of Labour and Social Protection*,* [the idea] then was for MOH and CoG to work on the tools that would then be sent to the counties for them to use to identify and then set up a database. And then the database would be merged to the social protection database (KII_Do_01).*

The program was designed to initially cover approximately one million indigent households, with planned expansion to 1.5 million in the subsequent year and eventual scale-up to five million households, in line with national poverty estimates indicating that about 5.2 million households were living below the poverty line. Premiums were based the flat contribution rate for informal sector households under the national UHC scheme (KES 500 per household per month). For the first phase (2020/21), the National Treasury allocated KES 6 billion (approximately USD 46.4 million) to finance premiums for one million households for one year. Coverage was structured at the household level, extending beyond the principal member to include spouses and children; based on the national average household size of 4.4, the design anticipated coverage of approximately 4.4 million individuals.

### De facto implementation and fidelity of the UHC indigent programme

Implementation of the UHC indigent programme diverged from its intended design due to intergovernmental contestation, gaps in national readiness, and constrained operational capacity. These factors reshaped beneficiary identification, enrolment, and purchasing arrangements, with implications for implementation fidelity.

### Intergovernmental authority and decentralisation of beneficiary identification

Although the programme was designed to apply a nationally standardised approach to identifying indigent households, county governments asserted control over beneficiary identification, citing the constitutional devolution of health functions. This resulted in a negotiated arrangement in which the national government allocated each county a fixed quota of indigent households, while counties retained discretion over identification methods:

*Midway there was a contestation… the national government set a cap for all the counties*,* and each of the counties were then allowed to identify households on their own* (KII_Do_01).

Resistance to national identification was also driven by political and economic interests. Specifically, county-level political actors were concerned that nationally led targeting would exclude them from the identification process, concentrating visibility and political credit at the national level. As a result, locally elected leaders would miss the opportunity to associate themselves with programme benefits or claim political mileage from subsidised coverage delivered in their regions:

*There is also a political economy dimension. Politicians wanted to be associated with these benefits*,* so if indigents were identified without their involvement*,* politically they would be left out. They wanted to be able to tell people that they are the ones who facilitated access to coverage. KII_IP_04*

Some stakeholders noted that a centrally driven indigent programme did not sufficiently account for pre-existing county subsidy schemes targeting poor households. Counties such as Makueni and Kisumu had already established programmes including MakueniCare and Marwa shortly after devolution, shaping local expectations around autonomy and programme ownership:

*Some programs came before this. MakueniCare started immediately after devolution… some counties started before the National Government (KII_C1_06).*

### Constraints in national targeting infrastructure and data governance

Decentralisation of identification was also partly due to gaps in national readiness at the time of rollout. Several county officials reported that the HTT was not finalised or operationalised, while access to MOLSP’s ESR was constrained by formal data-sharing requirements:

*The Ministry of Labor and Social Protection had the data*,* but there were challenges in sharing the information (KII_MOH_04).*

In some cases, respondents indicated that available national datasets in ESR were incomplete or insufficiently updated to meet county quotas within short timelines. These limitations increased reliance on locally generated lists.

### Localised targeting practices and erosion of household-level assessment

Counties received indigent household quotas based on population size and poverty levels from the 2019 census. To fill these quotas, counties adopted various identification practices, including community poverty ranking- where local actors identify households perceived to be most vulnerable based on community knowledge- and locally adapted proxy means testing, which estimates household welfare using simplified socioeconomic indicators tailored to local contexts. CHVs, village elders, and chiefs played central roles. While this flexibility allowed contextual adaptation, it produced substantial variation in eligibility criteria across counties:

*Once we decentralised the identification*,* I could qualify as an indigent in Nyeri*,* but not in Uasin Gishu (KII_Do_01).*

Under significant political and administrative pressure to meet quotas quickly, identification in some cases shifted away from household-level assessment toward public-space registration, including markets. This increased the likelihood of enrolling individuals with existing NHIF coverage:

*We were supposed to go to the household level… but now we were going to the markets and those who had NHIF were being registered (KII_C1_06).*

These practices blurred distinctions between indigent households and other priority groups, including older persons, people with chronic illness and/or disabilities, and CHVs who had been promised NHIF coverage under separate commitments. Overlaps with other subsidy programmes, such as those supporting orphans and vulnerable children, further increased the risk of duplication. Ultimately, counties reported difficulty identifying sufficient eligible households within the allotted timeframe:

*I was given twenty-eight thousand indigents… I ended up with eighteen thousand. Where do you get them from? (County official).*

### Community-level operational capacity constraints

Operational limitations at the community level compounded targeting challenges. CHVs reported uncertainty about eligibility criteria, limited logistical support, and reliance on paper-based data collection, slowing identification and verification:

*The data collection process was slow because we didn’t have proper devices for CHWs*,* and much of the work was done manually (KII_C1_04).*

Household data management required cleaning and validation of large volumes of submissions. Many counties lacked adequately trained personnel to perform these tasks efficiently:

*We didn’t have enough trained personnel to handle the amount of data coming in (KII_C1_03).*

In the absence of dedicated national funding to support CHV logistics, several counties relied on development and implementing partners to facilitate training, supervision, and data collection, resulting in uneven implementation capacity across counties.

### Verification and enrolment constraints

Once household lists were submitted, NHIF verified entries against national databases, including IPRS and MOLSP records. A substantial proportion of entries were excluded due to duplication, existing NHIF coverage, or inconsistencies with civil registration data. Counties received limited feedback on verification outcomes, constraining their ability to correct errors in subsequent submissions or communicate enrolment status to the households.

Eligible households were subsequently enrolled into Supa Cover, with premiums fully financed by the national government. According to an NHIF senior official, 882,291 households were registered from an initial submission of 1,509,037 households. The second phase of enrolment was characterised by extreme time pressure, which further weakened targeting processes, particularly in counties without pre-existing indigent databases. Counties were required to submit lists within 24 h during a festive period:

*The information came on 21st December and people were required to submit a list within 24 hours. KII_IP_04*

As a result, counties relied on rapid name collection rather than beneficiary identification, undermining confidence in the programme’s integrity. Subsequently, the Health Sector Medium-Term Expenditure Framework (2024/25–2026/27) reported that funds initially allocated for unregistered indigent households were redirected to provide insurance coverage for 200,000 boda boda riders. This reallocation was not formally communicated to counties, many of which had already submitted additional lists:

*We sent the second lists to NHIF*,* but after that*,* there was no follow-up (KII_C1_05).*

Even where enrolment was completed, communication gaps left many beneficiaries unaware of their registration status. In addition, dependents of household heads were not registered in many cases, which limited coverage for the entire household-the intended population unit. Biometric registration lagged behind enrolment:

*Biometric registration was delayed… and some households had difficulty accessing services (KII_Do_03).*

Only 382,000 individuals, (48% of the enrolled population), completed biometric registration, creating challenges for the remaining households when seeking services at healthcare facilities Table 5.

**Table 5:** UHC indigent program outcomes

Outcome	Target	Achieved	Completion Rate
Households enrolled	1,000,000	882,291	88%
Biometric Registration	1,000,000	382,000	48%

### Service access and facility empanelment

Under Supa Cover, outpatient services were financed through capitation, requiring empanelment of households to facilities of choice. However, for the indigents, Counties were given discretion in assigning public facilities, resulting in varied approaches. Some counties empanelled beneficiaries to nearby PHC facilities, while others restricted access to level 4 hospitals, influenced by higher capitation rates. In one study county, capitation funds were channelled through a level 5 referral hospital acting as a central disbursement facility. This interim arrangement was adopted to simplify fund flows where lower-level facilities lacked independent bank accounts:

*Funds for the UHC [indigent] programme are channelled to one facility… then distributed based on reported service use (KII_C2_02).*

Implementation was further shaped by county-level public financial management arrangements, particularly in counties where health funds were required to flow through the County Revenue Fund (CRF). In these settings, capitation and reimbursement payments from NHIF were first deposited into the CRF before being released to facilities, introducing additional administrative steps and delays. By contrast, counties that granted facilities greater financial autonomy- allowing NHIF funds to flow directly into facility accounts-reported more timely access capitation funds.

Delays in disbursement from the National Treasury to NHIF disrupted cash flow and undermined the timeliness of provider payments. As a result, NHIF delayed capitation and reimbursement to facilities, constraining their ability to procure medicines, pay operational costs, and sustain service delivery for beneficiaries.

*Funding would come late from the Treasury*,* so we’d delay reimbursements or capitation to facilities. That affected service delivery (KII_NHIF_02).*

Nonetheless, following the program’s implementation, some counties reported an increase in service utilization among indigent households, particularly in areas where biometric registration rates were higher.

*We saw a significant increase in utilization*,* especially among those who had completed biometric registration. Many were accessing services they had previously avoided due to cost* (KII_MOH_04).

### Stakeholder capacity

The implementation experiences described above point to differences in how responsibilities, resources, and authority were exercised across institutions involved in the UHC indigent programme. Table 6 summarises these patterns by presenting a comparative overview of the analytical, operational, and political capacities observed across key stakeholders during programme rollout.

**Table 6:** Stakeholder policy capacity

Stakeholder	Analytical Capacity	Operational Capacity	Political Capacity
**Ministry of Health (MOH)**	Developed overall policy framework but lacked the capacity to standardize data tools and processes across counties; limited engagement in data validation.	Provided policy guidance but had limited ability to enforce implementation fidelity after decentralization.	Strong national-level commitment to UHC, but limited influence over county-level political dynamics.
County Governments	Varied ability to analyse poverty data; frequently relied on subjective methods like community ranking without standardized tools.	Led household identification and enrolment but faced challenges in execution, especially in counties dependent on CRF with delayed fund flows of capitation from NHIF to the health facilities.	Held significant local influence over enrolment processes, which occasionally led to politicization and inconsistencies.
NHIF	Able to validate submitted data using national databases (e.g., IPRS) but depended on county-generated lists and lacked direct control over targeting criteria.	Effectively managed enrolment and benefits administration; however, struggled with delayed feedback loops and integrating non-standardized data.	Strong institutional mandate, but role constrained by upstream decentralization of beneficiary identification.
Ministry of Labour and Social Protection (MOLSP)	Maintained strong analytical tools and a national socioeconomic registry (ESR); capable of implementing proxy means testing (PMT).	Minimal involvement in program operations despite being the mandated custodian of social protection data.	Politically influential at national level but exhibited reluctance to collaborate fully, limiting integration with MOH and counties.
Community Health Volunteers (CHVs)	Limited training in data collection protocols; low familiarity with PMT tools and criteria.	Played a central role in household identification but lacked adequate resources (e.g., training transport, digital tools).	Minimal formal influence, but crucial for community engagement and trust-building.
Village Elders	Relied on local knowledge but used subjective criteria, leading to inconsistent identification practices.	Supported grassroots mobilization and enrolment; limited training and clarity on their role in verification.	Held informal authority in communities; facilitated access but lacked decision-making power.
Civil Society Organizations (CSOs)	Limited involvement in technical design or data analysis.	Focused on advocacy and community sensitization; not actively engaged in operational implementation.	Advocated for equity and inclusion but had limited leverage to influence policy direction.
Development Partners	Provided early technical assistance in designing policy frameworks; engagement reduced post-decentralization.	Supported system design and initial planning; limited involvement in day-to-day implementation.	Influenced agenda-setting at the national level but not embedded in domestic political processes.

### Broader policy context

At the time of rollout, multiple health insurance subsidy programmes targeting vulnerable groups were already in place. These included approximately 374,500 Inua Jamii beneficiaries enrolled through NHIF, about 45,000 households covered under county schemes such as Marwa in Kisumu, and additional coverage supported by development partners, including 22,000 refugee households and around 1,000 households sponsored by AMPATH. The coexistence of these programmes contributed to overlapping beneficiary pools and fragmented targeting arrangements.

### Unintended consequences of the UHC indigent program

A significant unintended consequence of the program was the presence of inclusion and exclusion errors in the identification of indigent households. Some households that were truly eligible for the program were left out, while others that did not qualify were included. This was largely due to discrepancies in data management and political interference at the county level.

*The biggest challenge was identifying the right people; we ended up with many people who were not truly indigent* (KII_C1_05).

These errors undermined the credibility of the program and limited its ability to reach the most vulnerable populations.

*We found that some households that were not truly indigent managed to get enrolled*,* while others that desperately needed the program were left out. This happened because the identification process wasn’t as rigorous as it should have been. (KII_IP_03)*.

Inconsistencies in the implementation process and the perception that the system was flawed also led to a breakdown of trust between communities and the institutions involved in delivering the program.

*[Inconsistencies]… meant that the trust in the process was a bit flawed in some communities. That is detrimental to any health system… [and] the blame would go to the institution involved in that process. So*,* I think for me*,* the biggest thing is that it resulted in mistrust around the process* (KII_IP_02).

Lastly, respondents highlighted stigma and concerns about dignity as an unintended consequence of the indigent programme, arguing that visible identification of households as “poor” discouraged participation and service use. The requirement to be explicitly classified as indigent was perceived to undermine social acceptability:

*The world need not know that I am a poor person.* KII_IP_04.

### Looking forward: views on the SHA beneficiary indigent program design

Stakeholders expressed mixed and often critical views regarding the planned use of PMT under SHA to assess household income and determine eligibility for subsidized coverage. According to an official from SHA, the PMT tool estimates household income based on responses to a standardized set of socioeconomic indicators, applying a statistical model akin to regression analysis.

*It [the PMT tool] estimates the household income based on the socioeconomic indicators in the questionnaire and then applies 2.75% of that income to determine the household premium. KII_NHIF_01*.

The decision to adopt PMT under SHA was partly influenced by lessons learned during the implementation of the UHC indigent program, where inconsistent and subjective targeting methods across counties posed major challenges.

*So*,* yes*,* there were challenges around identification [under the UHC indigent program]. That’s one of the reasons why there was a suggestion of developing a scientific model-that is*,* the proxy means testing-that can identify and quantify household income so that way it’ll reduce the subjectivity that was there and the variance across different counties.* (KII_NHIF_02)

The PMT tool applies context-specific questionnaire modules, with different indicator sets for rural, peri-urban, and urban households to reflect variation in livelihoods, asset ownership, and living conditions. Despite this contextual tailoring, several challenges were identified. These included the complexity of the questionnaire for individuals with low literacy, the risk of deliberate under-reporting of socioeconomic characteristics to reduce assessed contributions, and the time and resources required to roll out PMT across the informal sector, which accounts for approximately 83% of Kenya’s workforce. Concerns were also raised about the inclusiveness of means testing for the poorest households, many of whom lack mobile phones or the flexibility to attend assessment processes. As one county official cautioned:

*Indigents are people without a phone most of the time so you will not catch them… one day of not working means no food on that day*. Validation workshop participant 15

Other stakeholders questioned the underlying assumptions of the tool, pointing out that poverty is socially constructed and not easily captured through standardized indicators. *“Poverty at the local level is social*,* it is not mathematical*,*”* one county official explained, arguing that PMT fails to reflect nuanced household realities. Participants also raised concerns about potential manipulation of the PMT system, once its algorithm becomes widely known suggesting that the tool could be gamed, undermining its purpose. Stakeholders emphasized the need for greater transparency, community validation, and a reconsideration of whether PMT is the most appropriate method for targeting in Kenya’s informal and decentralized context.

*Kenyans… will congregate and give the lowest indicator*,* so that it gives you the lowest premium*, Validation workshop participant 19

Lastly, participants highlighted the political sensitivity of indigent identification under the Social Health Insurance (SHI) Act, particularly in relation to the co-financing responsibilities assigned to county governments. Respondents indicated that county willingness to allocate resources for indigent households would be closely tied to their involvement in, and ownership of, the identification process. Where beneficiary identification was perceived as externally driven or insufficiently inclusive of county leadership, respondents anticipated resistance at the implementation stage. A respondent described how governors might respond in such circumstances:

*If somebody has come to identify indigents in my county without me being involved… I will not use my money to pay for anyone. KII_IP_04*

## Discussion

This study examined the implementation experience of Kenya’s UHC indigent program to draw lessons for the ongoing rollout of the ongoing Social Health insurance reforms under SHA. Our findings reveal a significant disconnect between the intended centralized design and the decentralized execution of the program. Although the program aimed to leverage standardized national tools such as the MOLSP harmonized ing tool and socioeconomic registry, operational challenges, political conations, and limited stakeholder engagement led to widespread variation across counties. Reflections from the stakeholder validation workshop corroborated these findings, highlighting that decentralization was driven not only by county demands but also by the lack of readiness and financing at the national level to implement centralized tools. These dynamics undermined implementation fidelity and were consistent with findings from studies of Kenya’s devolved health system demonstrating how fragmented authority, limited enforcement capacity, and misaligned financing and information systems constrain the implementation of nationally designed programmes [9, 32].

The implementation experience was further characterized by institutional fragmentation and capacity mismatches across national and county stakeholders. While the MOH demonstrated political commitment to national UHC goals, its limited operational and analytical capacity hindered effective coordination and oversight. Similarly, while the NHIF managed enrolment processes, the process remained dependent on inconsistent county submissions, and the MOLSP, despite holding national datasets, operated parallel systems with limited willingness for data sharing. These dynamics were exacerbated at the county level, where reliance on under-trained CHVs (now known as community health promoters-CHPs), political expediency, and constrained fiscal systems led to inconsistent beneficiary identification and limited-service access.

Our findings build on and extend the evidence from Nyawira et al. [39], who examined the design and implementation experience of Kenya’s 2018 UHC pilot in four counties [39]. Their study highlighted persistent challenges such as unclear benefit packages, weak alignment between national and county financing mechanisms, delays in fund disbursement, and limited supply-side capacity. While these issues were similarly found in our analysis, our process evaluation of the national UHC indigent program (2020/21–2022) provides additional insights into how and why these challenges persisted at scale under a devolved governance context. Our study identifies the institutional and political drivers-particularly fragmented accountability, capacity mismatches, and shifting national priorities-that shaped implementation fidelity and equity outcomes. These findings therefore complement the pilot-phase evidence by explaining the dynamics of scale-up and the systemic barriers that must be addressed in future iterations under the SHA framework.

According to study respondents, utilization of health services in some facilities increased following the rollout of the indigent program, with households reporting improved access to outpatient and inpatient care. Similar patterns have been observed in sub-Saharan Africa, although evidence remains mixed. In Ethiopia, [38] found that community-based health insurance increased service utilization among vulnerable households in the Amhara region, while [10] reported no significant effect of a free health insurance program for the poor on utilization or financial protection in Senegal. Evidence from Ethiopia and Cambodia [20, 40] shows that even with free coverage, barriers such as limited awareness, transport costs, and perceived poor quality of care continue to constrain use. These findings suggest that while subsidized insurance improves financial access, its effectiveness among indigent populations also depends on addressing broader social and system-level barriers to care.

In Kenya’s UHC indigent program, several operational gaps hampered the realization of increased access for the poor. First, biometric registration was not completed for a significant proportion of enrolled households, limiting their ability to authenticate themselves and access services easily. Second, many dependents of registered household heads were not captured in the system, excluding significant portions of vulnerable families from coverage. Third, a critical gap was the lack of effective communication to beneficiaries regarding their entitlements under the program, resulting in missed opportunities to seek care even when financial barriers had been nominally removed [37]. These gaps demonstrate that enrolment alone is insufficient to guarantee access; effective delivery of subsidised coverage requires complete registration processes, inclusion of dependents, and sustained beneficiary awareness efforts.

Financing and purchasing arrangements further shaped how the programme was experienced at facility level. In several counties, capitation funds were channelled through higher-level facilities as an interim public financial management measure to facilitate fund flow. Higher capitation rates for level 4 and level 5 facilities created incentives for counties to empanel indigent beneficiaries at these facilities, even where lower-level facilities were available, as documented in previous analyses of the indigent programme [37]. In settings where level 4 and 5 facilities were located far from beneficiaries, this empanelment practice introduced additional non-financial barriers, including transport costs and travel time, thereby limiting effective access for poor households.

These findings are particularly salient as Kenya transitions to the SHA-led indigent programme. While the SHI Act centralises key functions-including enrolment, eligibility assessment, and fund management- under the SHA, the requirement for county co-financing preserves many of the decentralisation dynamics observed during the UHC indigent programme. As a result, tensions between national standardisation and county implementation capacity are likely to persist. Although the SHI Act envisaged the integration of proxy means testing (PMT) into enrolment processes, recent policy guidance indicates that the initial rollout of the indigent programme will rely primarily on existing administrative datasets, notably the Social Registry and the Inua Jamii cash transfer programme maintained by MOLSP, supplemented by SHA-led validation validation (Ministry of Health, 2025). It remains unclear whether this approach represents a transitional step or a substantive shift away from PMT-based identification as originally envisaged.

Our findings indicate that stakeholders expressed concerns about the accuracy and inclusiveness of both PMT, noting that poverty is often socially constructed and not fully captured through standardised indicators alone. This underscores the need for blended approaches that combine digital targeting tools with community-based validation mechanisms. A growing body of literature similarly highlights that assessments of vulnerability must account for social, cultural, and contextual dimensions of deprivation, which shape how poverty is experienced and recognised across settings [5, 18, 42]. Furthermore, relying on self-reported poverty assessment exposes the process to manipulation and ultimately inclusion and exclusion errors as has been found in Ghana’s indigent program where beneficiaries often falsified their socioeconomic status in order to benefit from exemption [2, 4, 28]. Without such integration, there is a risk of exclusion errors that could undermine the equity objectives of the SHA reforms. Emerging evidence suggests that advanced digital approaches, including machine-learning models trained on non-traditional data sources such as mobile phone usage, may complement existing tools and improve targeting accuracy if applied transparently [3].

Moreover, our findings highlight the importance of institutionalising feedback and learning mechanisms within programme implementation. The absence of structured channels for counties and NHIF to share verification outcomes meant that households were excluded from enrolment without explanations or opportunities for follow-up, contributing to mistrust at the community level and weakening programme credibility. Similar concerns have been documented in Ghana’s Livelihood Empowerment Against Poverty programme, where inclusion and exclusion errors generated perceptions of unfairness in beneficiary identification [2]. For SHA to strengthen public trust, grievance-handling and feedback mechanisms will need to be clearly defined and accessible at both facility and household levels. The SHI Act proposes the establishment of a tribunal to receive and resolve grievances tribunal [44]; however, key details-such as whether this mechanism will function at national or county level-remain unclear.

The role of stakeholder capacity-as assessed through the policy capacity framework-emerged as a key determinant of program fidelity. Future reforms must go beyond policy design to invest in building the analytical, operational, and political capacity of actors across levels. For example, CHPs and village elders need consistent training, supervision, and digital tools to support their role in community verification. Counties need technical and financial support to manage indigent enrolment in a way that is consistent with national guidelines but responsive to local realities. And inter-agency collaboration, especially between MOLSP, SHA, and county governments, must be incentivized through shared data systems, joint planning platforms, and aligned accountability frameworks.

Finally, the rollout of the new indigent program must reckon with the political economy of targeting. The UHC indigent program showed that political discretion at the local level influenced beneficiary selection, sometimes at odds with objective need. Understanding the political dynamics involved in expanding pro-poor policies is crucial for effectively scaling up and sustaining successful antipoverty programs. This includes considerations like the bargaining strength of beneficiaries, public support, and potential for political misuse of programs [12]. SHA must therefore ensure that targeting processes are both technically sound and governed by clear accountability arrangements, including mechanisms such as routine audits, transparent eligibility criteria, and alignment of county financing with agreed performance standards.

### Study strengths and limitation

This study is among the few to assess the implementation experience of Kenya’s UHC indigent program, providing an examination of both the de jure program design and de facto implementation realities. The main strength of the study is the integration of multiple data sources, including document reviews, semi-structured interviews with a diverse range of stakeholders across national and county levels, and validation of findings through a large stakeholder workshop attended by key actors involved in health financing reforms.

However, the study had some limitations. The absence of a consolidated national document detailing the design and implementation guidelines for the UHC indigent program necessitated reliance on stakeholder interviews and secondary reports to reconstruct the intended program design. This reliance may introduce recall bias and divergent interpretations among respondents. However, these lessons are important for informing the design, stakeholder engagement strategies, and accountability mechanisms necessary to strengthen Kenya’s social health insurance reforms and those of other LMICs pursuing similar UHC goals.

## Conclusion

This study reveals that while the UHC indigent program in Kenya was a well-intentioned step toward equitable health financing, its implementation was hindered by decentralization pressures, inadequate national readiness, and fragmented operational capacity. The resulting inconsistencies in targeting and enrolment compromised the program’s ability to fully protect poor households and foster trust. As Kenya embarks on implementing a new indigent coverage mechanism under the SHA, success will depend on addressing these foundational gaps. This includes improving data harmonization, ensuring full biometric and dependent enrolment, embedding grievance mechanisms, and enhancing stakeholder capacities at both national and county levels. A key lesson is the importance of blending standardized digital systems with community-level validation to reflect local understandings of poverty. As other LMICs design or refine similar subsidy programs, Kenya’s experience offers valuable insights into navigating the intersection of technical design, political economy, and implementation realities in the pursuit of universal health coverage. 

## Data Availability

Data available upon request.
